# COVID‐19 Information on YouTube: Analysis of Quality and Reliability of Videos in Eleven Widely Spoken Languages across Africa

**DOI:** 10.1155/2023/1406035

**Published:** 2023-01-18

**Authors:** Kapil Narain, Kingsley Appiah Bimpong, O’Neil Kosasia Wamukota, Oloruntoba Ogunfolaji, Udeme-Abasi U. Nelson, Anirban Dutta, Ayodeji Ogunleye, Eileen van der Westhuizen, Emmanuel Eni, Almthani Hamza Abdalrheem, Samuel Mesfin Girma, Aimée Bernice Munezero, Nazo Nxumalo, Okuhle Xozwa

**Affiliations:** ^1^ Federation of African Medical Students’ Associations COVID-19 Technical Working Group Ibadan, Nigeria; ^2^ Nelson R Mandela School of Medicine University of KwaZulu-Natal Durban, South Africa, ukzn.ac.za; ^3^ Department of Pediatrics and Child Health Tamale Teaching Hospital Tamale, Ghana, tamaleteachinghospital.org; ^4^ Maseno University Kisumu, Kenya, maseno.ac.ke; ^5^ College of Medicine University of Ibadan, Nigeria, ui.edu.ng; ^6^ University of Uyo Uyo, Nigeria, uniuyo.edu.ng; ^7^ University of Hawaii Honolulu HI, USA, hawaii.edu; ^8^ Obafemi Awolowo University Ile-Ife, Nigeria, oauife.edu.ng; ^9^ Bayero University Kano, Nigeria, buk.edu.ng; ^10^ Nile Valley University Faculty of medicine and Health Sciences Atbara, Sudan, upm.edu.my; ^11^ School of Medicine Addis Ababa University, Ethiopia, aau.edu.et; ^12^ University of Rwanda Kigali, Rwanda, ur.ac.rw; ^13^ Faculty of Health Sciences Walter Sisulu University Mthatha, South Africa, wsu.ac.za

## Abstract

**Introduction:**

Whilst the coronavirus disease 2019 (COVID‐19) vaccination rollout is well underway, there is a concern in Africa where less than 2% of global vaccinations have occurred. In the absence of herd immunity, health promotion remains essential. YouTube has been widely utilised as a source of medical information in previous outbreaks and pandemics. There are limited data on COVID‐19 information on YouTube videos, especially in languages widely spoken in Africa. This study investigated the quality and reliability of such videos.

**Methods:**

Medical information related to COVID‐19 was analysed in 11 languages (English, isiZulu, isiXhosa, Afrikaans, Nigerian Pidgin, Hausa, Twi, Arabic, Amharic, French, and Swahili). Cohen’s Kappa was used to measure inter‐rater reliability. A total of 562 videos were analysed. Viewer interaction metrics and video characteristics, source, and content type were collected. Quality was evaluated using the Medical Information Content Index (MICI) scale and reliability was evaluated by the modified DISCERN tool.

**Results:**

Kappa coefficient of agreement for all languages was *p* < 0.01. Informative videos (471/562, 83.8%) accounted for the majority, whilst misleading videos (12/562, 2.13%) were minimal. Independent users (246/562, 43.8%) were the predominant source type. Transmission of information (477/562 videos, 84.9%) was most prevalent, whilst content covering screening or testing was reported in less than a third of all videos. The mean total MICI score was 5.75/5 (SD 4.25) and the mean total DISCERN score was 3.01/5 (SD 1.11).

**Conclusion:**

YouTube is an invaluable, easily accessible resource for information dissemination during health emergencies. Misleading videos are often a concern; however, our study found a negligible proportion. Whilst most videos were fairly reliable, the quality of videos was poor, especially noting a dearth of information covering screening or testing. Governments, academic institutions, and healthcare workers must harness the capability of digital platforms, such as YouTube to contain the spread of misinformation.

## 1. Introduction

The World Health Organisation (WHO) declared coronavirus disease 2019 (COVID‐19) a public health emergency of international concern on 20 January 2020, and a global pandemic on 11 March 2020 [1]. As of 27 June 2022, there were over 544 000 000 cases and over 6 330 000 deaths due to COVID‐19 [2].

At the onset of the pandemic, the African response of instituting swift lockdown had been applauded [3]. However, WHO has cautioned that the actual number of COVID‐19 cases is far higher in Africa due to limited testing. This was despite the Partnership to Accelerate COVID‐19 Testing (PACT) initiative by the African Union Commission and the Africa Centers for Disease Control (Africa CDC), which aimed at improving testing capacity [4]. Officially, 8.5 million cases were reported for the African continent, though the WHO found that only 14.2% of cases were detected [5]. Furthermore, according to a report by the WHO in October 2021, 70 million tests have been conducted, a fraction of the continent’s population of 1.3 billion [5].

The epidemiological situation is further compounded by a poor vaccination plan. According to a recent analysis by the WHO, Africa scored 33% readiness for the rollout of the COVID‐19 vaccine, far below the required 80% benchmark [6]. Furthermore, vaccine inequity and nationalism have impeded vaccinations in Africa, with only 153.95 million doses administered compared with the global total of 10.34 billion as of 11 February 2022 [7]. This translates to less than 2% of global vaccinations occurring in Africa. Furthermore, adjusted for population, Africa has only received 48.31 doses per 100 people, whilst the global figure is 162.58 per 100. This is a concern noting that unvaccinated people have a higher risk of severe disease and admission. Furthermore, a recent study found that mortality amongst critically ill patients is higher in the African continent than in other continents; this occurrence is attributed to the lack of healthcare resources and comorbidities, including HIV/AIDS, and other chronic diseases [8].

The Internet is not only a key source of information for the public but also a catalyst of misinformation due to its capability of spreading information widely and rapidly [9]. According to Internet traffic estimates, as of December 2021, YouTube (a freely available, easy‐to‐use, and Internet video‐sharing platform with more than 2 billion users) was the second most popular website globally, with 1 billion hours of video watched daily [10, 11]. YouTube has been widely utilised as a source of medical information in previous outbreaks and pandemics [12–14]. Although the information found on this platform is questionable due to personal opinions, anecdotes, blind authorship, and a lack of credible sources, it is widely popular and trusted by viewers [9, 15].

A systematic review by Osman et al. of 202 articles assessing 22,300 health‐related YouTube videos found that about 41% of health‐related content objectively assessed using standard scoring systems across a wide range of medical specialties was reported to be inaccurate or “not useful” while only about 19% of such content was categorized as useful, with more than half reflecting commercial interests [16]. The same study also revealed that 44% of articles highlighted the role of verified health sources in promoting reliable information on YouTube with 11% discouraging the use of YouTube as a platform for promoting actionable health‐related content.

Google Trends, on 21 January 2022, indicated that the most searched topic on YouTube for the past 12 months, worldwide, was the coronavirus pandemic [17]. There have been a few studies evaluating the quality and reliability of COVID‐19 information on YouTube. Such studies report information as being suboptimal or poor, with over 25% of videos being misleading [18–20]. There remains a paucity of studies focusing on languages in Africa. In this study, the authors aim to evaluate the usefulness of YouTube as a source of COVID‐19 information in selected languages widely spoken across Africa, by characterizing the quality, reliability, viewer interaction metrics, source, type, and content of videos.

## 2. Methods

The methods used to carry out this work are explained in the following sections.

### 2.1. Languages

Videos in eleven languages (English, isiZulu, isiXhosa, Afrikaans, Nigerian Pidgin, Hausa, Twi, Arabic, Amharic, French, and Swahili) which are widely spoken or representative of all regions of Africa were evaluated.

### 2.2. Video Collection

**Figure 1:**
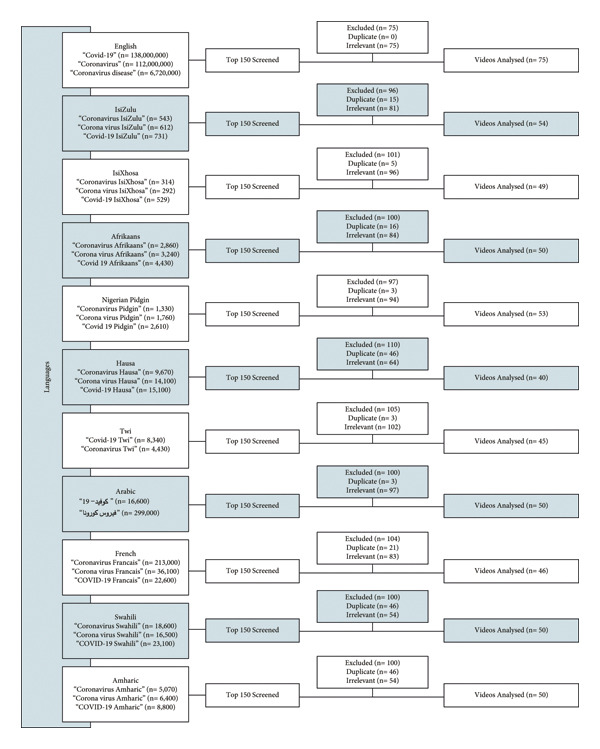
Data flow collection.

YouTube was accessed on 5 September 2020, using a new account with default settings, after clearing the cache of the Internet browser to minimize bias caused by cookies, personal preferences, and browser history. Combinations of search terms used included “coronavirus,” and “COVID‐19”, and attaching the name of the language thereafter as a specifier (Figure 1).

The first 50 relevant results were screened, and relevant videos that contained COVID‐19 medical or epidemiological information were included.

Videos concerning nonmedical information (that is socioeconomic impacts of lockdown or politics), of another language, without audio, or that were duplicated from previous search results were excluded.

After the initial 50 videos were screened, this process was repeated with the second and third search terms for a total of 50 videos for each language (with the exception of English for which 75 videos were included due to the higher volume of videos available). Figure 1 indicates the data collection flow. Cumulatively, 562 videos were marked for evaluation.

The final videos were saved on a playlist for evaluation, as search results on YouTube can change on a daily basis, and the Universal Resource Locators (URLs) of each video were captured [21]. This methodology is in accordance with previous studies [22–24]. Video characteristics such as the duration of the video (using the “mm: ss” format for minutes and seconds), upload date, interaction metrics (number of views, likes, dislikes, comments, and subscribers), video quality, and hashtags were all recorded at the time of video collection.

### 2.3. Evaluation

Videos of each language were scored by two independent viewers who had a medical background and were proficient in the respective languages. Kappa coefficient (*K*) was used to determine the degree of agreement between the two researchers for each language.

The reliability and quality of each video were assessed. The reliability was evaluated using a modified DISCERN tool [22, 25]. This tool comprises five questions assessed: clarity, reliable source, lack of bias, reference supplementation, and mention of uncertainty; each has “Yes” or “No” responses (Supplementary Table 1). “Yes” indicates good reliability, scoring 1 point, whilst “No” indicates poor reliability and scores 0 points. Consequently, each video can obtain a cumulative score between 0 (lowest reliability) and 5 (highest reliability).

Quality was assessed using the Medical Information Content Index (MICI) scale. This tool was previously devised for a study on the Ebola epidemic [21]. The MICI is a 5‐point Likert scale assessing five components of medical information: prevalence, transmission, clinical symptoms, screening/testing, and treatment/outcomes of disease. A maximum score of 25 was possible. Each component was graded utilising criteria adapted from a similar study evaluating the quality of COVID‐19 information on YouTube [18]. Guidelines from WHO and CDC were used for developing the criteria for the 5 components of the MICI scale (Supplementary Table 2).

The sources of videos were classified into one of the following groups: independent users, government/national agencies, news agencies, academic institutions/hospitals, and medical advertisements/for‐profit companies. The type of content was classified into four distinct groups: informative (factual information regarding prevention, screening, signs, and symptoms, testing, treatment, and epidemiology), misleading (scientifically inaccurate information or content that is not evidence‐based), personal (based on an individual’s experience or that of a friend or family), and news updates (content concerning statistical updates on cases, mortality, and recovery in the absence of providing information on symptoms, prevention, or management of COVID‐19). Furthermore, any incorrect or unscientific claims were documented.

### 2.4. Data Analysis

The like‐dislike ratio was calculated by dividing the number of likes by dislikes. Views per day were calculated by dividing the total number of views by the number of days that the video was uploaded for. Descriptive statistics were performed with means, standard deviation, median, and interquartile range. Categorical data were analysed using chi‐square tests and continuous data utilising ANOVA. A *p* value of 0.05 was considered significant. Data were analysed using R Studio (version 3.6.3, Vienna, Austria).

## 3. Results

The Kappa coefficient of agreement between the researchers for all eleven languages was statistically significant (*p* < 0.001), Table 1.

**Table 1:** Kappa coefficient of languages included in the study.

Languages	Number of videos	Kappa (*k*)	*p* value
English	75	0.62	<0.001
isiZulu	54	0.49	<0.001
isiXhosa	49	0.82	<0.001
Afrikaans	50	0.73	<0.001
Nigerian Pidgin	53	1.00	<0.001
Hausa	40	0.93	<0.001
Twi	45	1.00	<0.001
Arabic	50	0.82	<0.001
French	46	0.44	<0.001
Swahili	50	0.51	<0.001
Amharic	50	1.00	<0.001

### 3.1. Viewer Interaction Metrics

A total of 562 videos were included for analyses, with a cumulative total number of 277,708,221 views (range, 0–35,311,150), 4,626,536 (range, 0–910 000) likes and 375 323 (range, 0–54 000) comments (Table 2). The median number of views per day was 2.4 (IQR, 0.4–364.9), the median duration was 2 minutes and 48 seconds (IQR: 1 : 45–4 : 58), and the median like/dislike ratio was 12.6 (IQR, 7–22.6) (Table 2). More than a quarter of all videos had more than 50 000 views and 506/562 (90%) videos were available in high definition.

**Table 2:** Viewer interaction metrics of YouTube videos analysed by language, video content, and source.

Characteristic	Number of videos	Total number of views	Median number of views (IQR)	Median number of views per day (IQR)	Median duration of videos in mm: ss (IQR)	Median like: dislike ratio (IQR)	Median number of comments (IQR)	Median number of subscribers (IQR)	Videos with views <100 no (%)	Videos with views> 50000 no (%)	High definition videos no (%)
Total	562	277,708,221	320 (32–48,545)	2.4 (0.4–364.9)	2 : 48 (1 : 45–4 : 58)	12.6 (7–22.6)	1 (0–49)	1220 (56–134,500)	209 (37.2)	140 (24.9)	506 (90)
*Languages*											
English	75	183,607,655	789092 (199548–2467314)	8198 (3261–22125)	6 : 31 (4 : 36–11 : 50)	15 (9.5–29.4)	2050 (761–5050)	964 500 (387,000–2,730,000)	0 (0)	75 (100)	75 (100)
isiZulu	54	128,092	35 (12–273)	0.4 (0.1–1.8)	2 : 16 (2 : 0–3 : 25)	7 (5.5–45.6)	0 (0–0)	51 (19–583)	35 (64.8)	3 (5.6)	54 (100)
isiXhosa	49	6,895	16 (11–78)	0.3 (0.1–0.6)	2 : 28 (1 : 58–3 : 28)	2.65 (1.8–3.5)	0 (0–0)	56 (54–262)	40 (81.6)	0 (0)	49 (100)
Afrikaans	50	37,121	82.5 (32–275)	0.9 (0.5–1.9)	2 : 08 (1 : 49–2 : 38)	3.4 (1.5–3.85)	0 (0–0)	56 (29–111)	28 (56)	0 (0)	31 (62)
Nigerian Pidgin	53	426,625	58 (20–249)	0.62 (0.2–2)	1 : 54 (1 : 0–3 : 18)	5.5 (3.3–23.7)	0 (0–1)	84.5 (30–168)	29 (54.7)	1 (1.9)	34 (64)
Hausa	40	339,120	533.5 (57–4902)	4 (0.4–30)	3 : 20 (1 : 9–4 : 40)	18 (11–24.3)	1 (0–5)	89 (53–162)	12 (30)	6 (15)	40 (100)
Twi	45	67,045	70 (15–279)	0.6 (0.2–1.6)	2 : 39 (1 : 53–3 : 43)	33.2 (23.6–60.5)	0 (0–2)	35 (11–130)	26 (57.8)	2 (4.4)	27 (60)
Arabic	50	72,755,229	213,102 (60,239–809,166)	1473 (353–5551)	3 : 23 (2 : 0–4 : 45)	11.65 (9.3–16.3)	97 (32–488)	1 920 000 (18400–6890000)	0 (0)	42 (84)	50 (100)
French	46	15,830,597	7307 (324–109679)	57 (2–600)	2 : 03 (1 : 7–3 : 43)	12.4 (6–17.9)	8 (1–181)	37000 (900–181000)	7 (15.2)	22 (47.8)	46 (100)
Swahili	50	4,148,448	317 (73–7825)	3 (1–63)	3 : 03 (1 : 39–4 : 47)	9.75 (5.6–15.1)	0 (0–5)	1220 (131–54050)	15 (30)	10 (20)	50 (100)
Amharic	50	361,394	305.5 (56–868)	2 (1–6)	4 : 06 (1 : 30–11 : 49)	20.5 (2.3–47.7)	0 (0–3)	5410 (3190–5410)	17 (34)	4 (8)	50 (100)
*Content*											
Informative	471	229,705,869	222 (26–7035)	1.7 (0.3–52)	2 : 45 (1 : 45–4 : 51)	13.4 (7.7–25.7)	0 (0–15)	307 (51–26500)	192 (40.8)	110(23.4)	419 (88.96)
Misleading	12	75,172	280.5 (78–5306)	1.7 (0.6–27.48)	1 : 53 (1 : 14–3 : 42)	49.15 (32.5–66.9)	6 (0.5–8.5)	133 (19–679)	4 (33.3)	2 (16.7)	9 (75)
News update	59	41,144,689	88837 (7669–493260)	1050 (55–7409)	3 : 13 (1 : 56–6 : 33)	8.95 (5.5–13.6)	90 (1–831)	2260000 (447500–6890000)	8 (13.6)	43 (72.9)	58 (98.31)
Personal experience	20	6,782,491	8549.5 (106–547955)	50 (0.6–4954)	3 : 05 (2 : 1–7 : 43)	16.95 (9.7–21.0)	148 (0.5–3150)	5560 (895–135500)	5 (25)	10 (50)	20 (100)
*Source*											
Independent users	246	108,094,174	271.5 (31–9519)	1.8 (0.3–71.2)	3 : 12 (2 : 1–5 : 42)	14 (7.2–26.4)	0.5 (0–54.3)	164 (25–13600)	92 (37.4)	61 (24.8)	218 (88.62)
Government	84	15,273,139	102 (24–1199.75)	1.1 (0.3–8.8)	2 : 01 (1 : 6–4 : 2)	9.15 (5.2–12.2)	0 (0–1)	5410 (152–8256)	42 (50)	10 (11.9)	80 (95.24)
News agency	118	81,800,404	41207 (544–387864)	296.3 (7.5–5714.2)	3 : 04 (1 : 39–5 : 3)	11.6 (6.7–17.9)	13.5 (1–651.3)	1920000 (311000–6890000)	14 (11.9)	67 (56.8)	113 (95.76)
Academic institutions/hospitals	93	70,931,103	59 (18–8867)	0.7 (0.2–63.8)	2 : 24 (1 : 54–4 : 35)	19.25 (11.8–34.4)	0 (0–7)	89 (56–27200)	52 (55.9)	23 (24.7)	81 (87.1)
Medical advertisements/for‐profit companies	21	1,609,401	115 (36–617)	1.2 (0.5–5.5)	2 : 25 (1 : 18–3 : 15)	10.3 (7–13.1)	0 (0–1.5)	162 (83–11100)	9 (42.9)	4 (19)	14(66.67)

Videos in English had the highest total number of views (183 607 655), followed by Arabic (72 755 229) and French (15 830 597), while IsiXhosa (6 895), Afrikaans (37 121), and Twi (67 045) had the lowest. Similarly, English had the highest number of median views (789 092), views per day (8 198), comments (2 050), and subscribers (964 500).

The majority of videos were classified as informative (471/562, 83.8%) with over 229 million views, while misleading videos constituted 12/562 (2.13%) of total videos, with a total number of views at over 75,000. News updates had the highest median number of views at 88,837 (7 669–493 260), while informative videos had the least at 222 (26–7305). Furthermore, misleading videos had the highest like: dislike ratio at 49.15 (32.5–66.9), while news updates had the least at 8.95 (5.5–13.6).

Independent users (246/562, 43.8%) comprised the majority of videos with over 100 million total views. Government videos had the least like: dislike ratio at 9.15 (5.2–12.2), whilst academic institutions had the most at 19.25 (11.8–34.4).

### 3.2. Distribution of Content and Source

Informative videos were the predominant content type across all languages comprising more than 70% of all videos with the exception of Arabic (54%). Misleading videos were minimal and found in isiZulu (1/54, 2%), Afrikaans (2/50, 4%), Nigerian Pidgin, and Hausa (both 3/50, 6%) (Table 3). Independent users were the majority source type across most languages, with the exception of English, Hausa, and Arabic (where news agency videos were predominant) and IsiXhosa, where academic institutions were the main source type. Adverts do not constitute the major source type in any language.

**Table 3:** Distribution of the content and source type across language.

Contents	English (*n* = 75)		isiZulu (*n* = 54)	isiXhosa (*n* = 49)	Afrikaans (*n* = 50)	Nigerian Pidgin (*n* = 53)	Hausa (*n* = 40)	Twi (*n* = 45)	Arabic (*n* = 50)	French (*n* = 46)	Swahili (*n* = 50)	Amharic (*n* = 50)
*Overall content no (%)*												
Informative		53(70.1)	47 (87)	45 (91.8)	46 (92)	42 (79.3)	33 (82.5)	43 (95.6)	27 (54)	38 (82.6)	47 (94)	50 (100)
Misleading		0 (0)	1 (2)	0 (0)	2 (4)	3 (6)	3 (6)	1 (2)	0 (0)	2 (4)	0 (0)	0 (0)
News update		17 (34)	0 (0)	1 (2)	0 (0)	8 (16)	4 (8)	0 (0)	21 (42)	5 (10)	3 (6)	0 (0)
Personal experience		5 (10)	6 (12)	3 (6)	2 (4)	0 (0)	0 (0)	1 (2)	2 (4)	1 (2)	0 (0)	0 (0)

*Source no (%)*												
Independent users		22 (29)	36 (67)	13 (27)	27 (54)	25 (47)	12 (30)	25 (56)	16 (32)	20 (43)	23 (46)	27 (54)
Government		5 (7)	3 (6)	15 (31)	5 (10)	2 (4)	4 (10)	4 (9)	5 (10)	11 (24)	9 (18)	21 (42)
News agency		27 (36)	1 (2)	2 (4)	1 (2)	16 (30)	19 (48)	3 (7)	29 (58)	7 (15)	13 (26)	0 (0)
Academic institutions/hospitals		21 (28)	13 (24)	19 (39)	14 (28)	6 (11)	5 (13)	7 (16)	0 (0)	3 (7)	5 (10)	0 (0)
Medical advertisements/for‐profit companies		0 (0)	1 (2)	0 (0)	3 (6)	4 (8)	0 (0)	6 (13)	0 (0)	5 (11)	0 (0)	2 (4)

### 3.3. MICI: Quality

Overall, information on the transmission of COVID‐19 was most prevalent (477/562 videos, 84.9%), whilst content covering screening or testing was least reported with less than a third of videos containing such information (Table 4 and Supplementary Table 2). The mean total MICI score was 5.75/25 (SD 4.35).

**Table 4:** MICI score by language, content, and source.

MICI item	Prevalence		Transmission		Clinical symptoms		Screening testing		Treatment/outcomes		Total
	Videos with information no (%)	MICI score mean ± SD	Videos with information no (%)	MICI score mean ± SD	Videos with information no (%)	MICI score mean ± SD	Videos with information no (%)	MICI score mean ± SD	Videos with information no (%)	MICI score mean ± SD	MICI score mean ± SD
Overall	249 (44.3)	0.98 ± 1.5	477 (84.9)	2.13 ± 1.4	352 (62.6)	1.52 ± 1.54	163 (29.0)	0.45 ± 0.84	246 (43.8)	0.68 ± 0.96	5.75 ± 4.35

*Languages*											
*p*	<0.001	<0.001	<0.001	<0.001	<0.001	<0.001	<0.001	<0.001	<0.001	<0.001	
English (*n* = 75)	32 (42.7)	0.89 ± 1.24	51 (68)	1.71 ± 1.62	38 (50.7)	1.56 ± 1.9	24 (32)	0.64 ± 1.22	42 (56)	0.97 ± 1.09	5.77 ± 4.5
isiZulu (*n* = 54)	20 (37)	0.44 ± 0.72	43 (79.6)	2.37 ± 1.57	29 (53.7)	1.76 ± 2.04	10 (18.5)	0.31 ± 0.8	15 (27.8)	0.67 ± 1.26	5.56 ± 4.52
isiXhosa (*n* = 49)	14 (28.6)	0.33 ± 0.55	45 (91.8)	2 ± 1.29	31 (63.3)	1.45 ± 1.39	10 (20.4)	0.37 ± 0.83	6 (12.2)	0.22 ± 0.74	4.37 ± 3.2
Afrikaans (*n* = 50)	10 (20)	0.22 ± 0.46	45 (90)	2.36 ± 1.06	30 (60)	1.18 ± 1.26	9 (18)	0.38 ± 0.95	20 (40)	0.56 ± 0.79	4.7 ± 2.37
Nigerian pidgin (*n* = 53)	14 (26.4)	0.36 ± 0.68	42 (79.2)	1.66 ± 1.27	30 (56.6)	1 ± 1.02	4 (7.5)	0.09 ± 0.35	14 (26.4)	0.32 ± 0.58	3.43 ± 2.56
Hausa (*n* = 40)	40 (100)	2.17 ± 0.81	40 (100)	2.62 ± 0.77	40 (100)	2.85 ± 0.83	40 (100)	1.6 ± 0.5	40 (100)	1.43 ± 0.5	10.68 ± 1.58
Twi (*n* = 45)	8 (17.8)	0.18 ± 0.39	42 (93.3)	2.11 ± 1.13	18 (40)	1.11 ± 1.53	1 (2.2)	0.02 ± 0.15	4 (8.9)	0.16 ± 0.52	3.58 ± 2.86
Arabic (*n* = 50)	20 (40)	0.56 ± 0.91	34 (68)	1.5 ± 1.37	32 (64)	1.48 ± 1.5	7 (14)	0.18 ± 0.48	23 (46)	0.56 ± 0.73	4.28 ± 2.96
French (*n* = 46)	19 (41.3)	0.54 ± 0.78	40 (87)	1.78 ± 1.25	25 (54.3)	1.2 ± 1.24	5 (10.9)	0.28 ± 0.86	17 (37)	0.78 ± 1.19	4.59 ± 3.54
Swahili (*n* = 50)	22 (44)	0.6 ± 0.81	45 (90)	1.48 ± 0.95	29 (58)	1.36 ± 1.4	3 (6)	0.12 ± 0.52	15 (30)	0.38 ± 0.64	3.94 ± 2.92
Amharic (*n* = 50)	50 (100)	4.68 ± 1.1	50 (100)	4.06 ± 0.24	50 (100)	1.96 ± 1.44	50 (100)	1.04 ± 0.28	50 (100)	1.36 ± 1.05	13.1 ± 2.95

*Content*											
*p*	<0.001	0.02	<0.001	<0.001	0.004	0.06	0.98	0.98	0.88	0.55	
Informative (*n* = 471)	208 (44.2)	1.02 ± 1.58	424 (90)	2.33 ± 1.35	309 (65.6)	1.59 ± 1.54	136 (28.9)	0.45 ± 0.85	208 (44.2)	0.7 ± 1	6.1 ± 4.42
Misleading (*n* = 12)	5 (41.7)	0.5 ± 0.67	10 (83.3)	1.83 ± 1.27	5 (41.7)	1 ± 1.35	3 (25)	0.5 ± 0.9	5 (41.7)	0.67 ± 0.89	4.5 ± 4.42
News update (*n* = 59)	35 (59.3)	1.02 ± 1.04	34 (57.6)	1.05 ± 1.21	31 (52.5)	1.22 ± 1.46	18 (30.5)	0.42 ± 0.72	26 (44.1)	0.53 ± 0.68	4.24 ± 3.3
Personal experience (*n* = 20)	1 (5)	0.05 ± 0.22	9 (45)	0.6 ± 0.75	7 (35)	0.95 ± 1.64	6 (30)	0.5 ± 0.95	7 (35)	0.55 ± 0.89	2.65 ± 3.36

*Source*											
*p*	0.06	<0.001	<0.001	<0.001	0.63	0.04	0.04	0.06	0.33	0.08	
Independent users (*n* = 246)	104 (42.3)	0.96 ± 1.56	215 (87.4)	2.26 ± 1.38	156 (63.4)	1.6 ± 1.62	61 (24.8)	0.35 ± 0.72	97 (39.4)	0.65 ± 1.03	5.81 ± 4.68
Government (*n* = 84)	38 (45.2)	1.51 ± 2.08	78 (92.9)	2.51 ± 1.43	53 (63.1)	1.15 ± 1.17	36 (42.9)	0.57 ± 0.84	38 (45.2)	0.55 ± 0.68	6.3 ± 4.59
News agency (*n* = 118)	65 (55.1)	1.04 ± 1.18	81 (68.6)	1.44 ± 1.28	67 (56.8)	1.36 ± 1.46	35 (29.7)	0.45 ± 0.77	59 (50)	0.64 ± 0.75	4.94 ± 3.96
Academic institutions/hospitals (*n* = 93)	33 (35.5)	0.54 ± 0.89	84 (90.3)	2.19 ± 1.34	62 (66.7)	1.82 ± 1.71	26 (28)	0.61 ± 1.16	44 (47.3)	0.92 ± 1.2	6.09 ± 3.84
Medical adverts/for profit companies (*n* = 21)	9 (42.9)	0.62 ± 1.12	19 (90.5)	2.52 ± 1.33	14 (66.7)	1.62 ± 1.4	5 (23.8)	0.43 ± 0.87	8 (38.1)	0.62 ± 0.92	5.81 ± 3.25

The highest total MICI score was attained by Amharic and Hausa (13.1/25 and 10.68/25, respectively) whilst Nigerian Pidgin, Twi, and Amharic had total scores below 4/25, and consequently, the lowest scores of all languages were analysed. All *p* values for MICI scores by language were statistically significant (*p* < 0.001).

Informative videos had the highest total MICI score (6.1/25) and, consequently, the highest score for all MICI indicators, aside from screening/testing, where personal experience and misleading videos reported the highest score. The personal experience videos had the lowest MICI score (2.65/25).

Government videos had the highest score for prevalence and transmission (tied with medical adverts), and the highest proportion of information pertaining to transmission and screening/testing. Academic institutions/hospitals reported the highest scores for clinical symptoms, screening/testing, and treatment/outcomes, but the lowest scores were for prevalence. News agencies reported the lowest score for transmission and the lowest proportion of videos covering transmission and clinical symptoms. Stratified by source, the highest total score was attained by government (6.3 ± 4.59) and academic institutions/hospitals (6.09 ± 3.84), whilst news agencies had the lowest (4.94 ± 3.96).

### 3.4. DISCERN: Reliability

The mean total DISCERN score was 3.01 ± 1.11 out of a possible five (Table 5 and Supplementary Table 2). The highest DISCERN item scores overall were reported on Item 1 (regarding whether video aims are clear and achieved), 0.97 ± 0.18, and Item 3 (regarding if the information was unbiased and unbalanced), 0.91 ± 0.28, and the lowest was Item 5 (whether any areas if uncertainty were mentioned), 0.25 ± 0.44. All DISCERN items were statistically significant across language, while DISCERN items 1–3 were statistically significant across content, and all DISCERN items aside from Item 5 were statistically significant across sources.

**Table 5:** DISCERN score by language, content, and source.

	DISCERN item 1 score mean ± SD	DISCERN item 2 score mean ± SD	DISCERN item 3 score mean ± SD	DISCERN item 4 score mean ± SD	DISCERN item 5 score mean ± SD	Total DISCERN score mean ± SD
Overall	0.97 ± 0.18	0.52 ± 0.50	0.91 ± 0.28	0.36 ± 0.48	0.25 ± 0.44	3.01 ± 1.11

Languages	<0.01	<0.001	<0.001	<0.001	<0.001	
English (*n* = 75)	1 ± 0	0.75 ± 0.44	0.99 ± 0.12	0.69 ± 0.46	0.44 ± 0.5	3.87 ± 0.91
isiZulu (*n* = 54)	0.91 ± 0.29	0.59 ± 0.5	0.78 ± 0.42	0.26 ± 0.44	0.13 ± 0.34	2.67 ± 1.37
isiXhosa (*n* = 49)	0.94 ± 0.24	0.53 ± 0.5	1 ± 0	0.18 ± 0.39	0.08 ± 0.28	2.73 ± 0.88
Afrikaans (*n* = 50)	0.9 ± 0.3	0.36 ± 0.48	0.8 ± 0.4	0.1 ± 0.3	0.08 ± 0.27	2.24 ± 1.06
Nigerian pidgin (*n* = 53)	1 ± 0	0.21 ± 0.41	0.98 ± 0.14	0.32 ± 0.47	0.17 ± 0.38	2.68 ± 0.85
Hausa (*n* = 40)	0.92 ± 0.27	0.82 ± 0.38	0.55 ± 0.5	0.6 ± 0.5	0.55 ± 0.5	3.45 ± 0.99
Twi (*n* = 45)	1 ± 0	0.33 ± 0.48	0.98 ± 0.15	0.22 ± 0.42	0.16 ± 0.37	2.69 ± 0.95
Arabic (*n* = 50)	0.98 ± 0.14	0.64 ± 0.48	0.96 ± 0.2	0.3 ± 0.46	0.36 ± 0.48	3.24 ± 0.94
French (*n* = 46)	0.98 ± 0.15	0.83 ± 0.38	0.96 ± 0.21	0.3 ± 0.47	0.26 ± 0.44	3.33 ± 0.87
Swahili (*n* = 50)	1 ± 0	0.26 ± 0.44	0.94 ± 0.24	0.48 ± 0.5	0.26 ± 0.44	2.94 ± 1
Amharic (*n* = 50)	1 ± 0	0.32 ± 0.47	1 ± 0	0.42 ± 0.5	0.28 ± 0.45	3.02 ± 1.24

Content	<0.001	<0.001	<0.001	0.48	0.10	
Informative (*n* = 471)	0.98 ± 0.14	0.53 ± 0.5	0.93 ± 0.26	0.37 ± 0.48	0.24 ± 0.42	3.03 ± 1.06
Misleading (*n* = 12)	0.75 ± 0.45	0.25 ± 0.45	0.58 ± 0.51	0.17 ± 0.39	0.25 ± 0.45	2 ± 1.35
News update (*n* = 59)	0.97 ± 0.18	0.61 ± 0.49	0.98 ± 0.13	0.41 ± 0.5	0.37 ± 0.49	3.34 ± 1.01
Personal experience (*n* = 20)	0.85 ± 0.37	0.1 ± 0.31	0.55 ± 0.51	0.35 ± 0.49	0.35 ± 0.49	2.2 ± 1.58

Source	0.02	<0.001	<0.001	0.25	<0.001	
Independent users (*n* = 246)	0.94 ± 0.23	0.37 ± 0.48	0.85 ± 0.35	0.32 ± 0.47	0.24 ± 0.43	2.73 ± 1.18
Government (*n* = 84)	1 ± 0	0.52 ± 0.5	1 ± 0	0.42 ± 0.5	0.11 ± 0.31	3.05 ± 0.97
News agency (*n* = 118)	0.97 ± 0.18	0.56 ± 0.5	0.91 ± 0.29	0.38 ± 0.49	0.41 ± 0.49	3.22 ± 1.1
Academic institutions/hospitals (*n* = 93)	1 ± 0	0.83 ± 0.38	0.97 ± 0.18	0.43 ± 0.5	0.24 ± 0.43	3.46 ± 0.88
Medical adverts/for‐profit companies (*n* = 21)	1 ± 0	0.57 ± 0.51	1 ± 0	0.29 ± 0.46	0.19 ± 0.4	3.05 ± 0.92

Across contents, news updates had the highest DISCERN scores for all items aside from Item 1, where informative videos had the highest score. Misleading videos had the lowest score for most DISCERN Items aside from Item 2 (a reliable source of information used) where personal experience had the lowest. Misleading and personal experiences had the lowest score for Item 3. Informative and misleading videos had the lowest score for item 5. The highest total score across content was achieved by news updates, whilst misleading videos had the lowest score.

Across sources, the highest total DISCERN score was obtained by videos from academic institutions/hospitals (3.46 ± 0.88), whilst the lowest was from independent users (2.73 ± 1.18). Items 1 and 3 had full or close to full scores across the sources.

## 4. Discussion

Videos in English recorded the highest number of views, followed by Arabic and French. This is not surprising, as these languages are among the most widely spoken languages in the world [26]. A previous study on YouTube, as a source of information for COVID‐19, showed that the English language has the second highest number of views, after the Hindi language [27].

The majority (83.8%) of the videos that were studied were informative, with misleading videos constituting only 2%. This was encouraging, as YouTube has become a major means of gaining information during pandemic periods [12, 13]. The sharing of misleading information on YouTube about the COVID‐19 pandemic has been a major challenge, as reported in a number of studies. Li et al. reported over a quarter of the viewed content containing misleading information [20]. Khatri et al. reported 10% and 8% of misleading videos, respectively, in their studies [18, 27]. The lower percentage seen in this study could be potentially accounted by the timing as it was conducted later than the previous studies, providing the opportunity for more robust infodemic curtailing measures. Gallotti et al. noted that social media information improved with an increase in infections [28].

Despite their modest number, misleading videos in our study had the highest likes: dislike ratio, whilst videos from government sources had the lowest. Similarly, misleading videos were proportionally liked five times more than their government counterparts. This phenomenon may be attributed due to the sensational nature of misleading videos, which may encourage individuals to like and share such content. Furthermore, the decreased digital support from users for government videos suggests a lack of confidence and trust in society. This is supported by a report that indicated that most African citizens did not trust their governments to provide accurate information about case counts and mortalities during this pandemic [29].

Our study revealed that independent users were the predominant source of information on the pandemic. These findings are supported by a previous study that evaluated YouTube as a source of information for the West Nile virus [14]. This is expected noting that YouTube is content‐driven and content creators on YouTube are paid for views [30]. However, YouTube has been identified as a major source of health information with a broad viewership. In view of this, professional bodies and healthcare organizations have been implored to embrace this technology for the sharing of medical information [18, 20, 31, 32]. IsiXhosa was the only language, which had the most (39%) of its content coming from academic institutions or hospitals. This may be an incidental, with no studies available to corroborate this finding.

Overall, across all languages, content on the transmission was the most reported (84.9%). This finding was not different from the study by Khatri et al. [18]. Content from screening or testing was the least reported, and even when this category of information was present, it was of poor quality indicated by low MICI scores. This was corroborated by other studies by Khatri et al. [18], Dutta et al. [27], and Ataç et al. [33]. With the advent of variants as identified in South Africa, low‐quality information on the technical aspect of the pandemic is worrying [34].

Overall, videos scored poorly for the quality of treatment information present. Brandi Ramos et al. noted that although videos on possible treatments for COVID‐19 had problems with quality, they had high viewer rates [35]. This further underscores the grave challenge experienced with sensationalism. This is particularly disturbing due to the promotion of unproven treatments, noting earlier reports of chloroquine poisoning in attempts to resort to this as treatment in Nigeria [35]. Furthermore, the falsehood of ivermectin as a cure continues to perpetuate as well as vaccine misinformation driving hesitancy, despite the evidence.

Government/national agencies and academic institutions/hospitals had the highest and second‐highest MICI scores, respectively. Nagpal et al. opined that reliable medical information with higher MICI scores could be provided by academic centres, government, and news agencies, and Singh et al. recommended that these institutions utilise YouTube as a platform for the dissemination of medical information [31, 36]. In our study, videos from these sources scored the highest among the various sources. However, the absolute MICI scores were still poor. This further emphasizes the potential for improvement with respect to the quality of the content of the videos produced by these sources. Videos from news agencies had the overall lowest MICI scores. Andika et al. reported that news agencies had greater odds of uploading useful videos compared to other sources [36]. This finding suggests that news agencies need to improve the quality of the information shared; particularly, information pertaining to treatment/outcomes is lacking.

The overall DISCERN score of 3.01 ± 1.11 suggests that most videos across all languages were fairly reliable. Similar findings were reported by Khatri et al. [18]. The presence of videos from academic institutions and hospitals were encouraging and in contrast to Nagpal et al. where these institutions did not contribute to uploading the videos regarding the Ebola epidemic at the time [32]. In this current pandemic, it was demonstrated that videos by healthcare professionals are more reliable than those of independent users [37, 38]. Szmuda et al. also noted that videos uploaded by physicians were the most reliable [39].

### 4.1. Recommendations

Several approaches have been implemented internationally and regionally in Africa to curb the infodemic in the setting of the COVID‐19 pandemic. Governments of various countries held press briefings to give accurate COVID‐19 information and combat fake news [40]. In Ghana, the Ministry of Information regularly issued flyers and press briefings to constantly educate and inform the public about the COVID‐19 situation in the country [41]. South Africa has utilised traditional and social media to frequently dispel myths and encourage vaccination uptake. Government and nonprofit organizations in countries such as Benin, Nigeria, and Sierra Leone have used platforms, such as WhatsApp, to disseminate information about the pandemic [42]. Furthermore, response and adherence to the public health interventions in Africa are usually marred by mistrust in the government alongside their perceived poor performing health systems [43, 44]. This makes content of the mainstream social media, YouTube, in particular, which is largely contributed to by independent users, a generally more acceptable source of information to the public.

Several measures should be taken to improve the quality and reliability of the information on YouTube. Firstly, public health agencies should collaborate with various YouTube content producers, particularly, influential independent users [20]. This will not only minimize the spread of misinformation but also attract viewership from a wider audience. Secondly, a qualified organization should be established and given the mandate to vet health‐related videos before they are uploaded on YouTube [45]. Such an organization would help flag down both the poor quality videos and those with inaccurate information. Lastly, similar to the peer review process with journal articles, health‐related YouTube videos should be subjected to rapid review by the health experts [46]. The health experts will ascertain the validity and reliability of the information contained in those videos; thus, ensuring high‐quality evidence based‐information and preventing misinformation from being viewed and shared by consumers.

### 4.2. Strengths and Limitations

This study has several strengths and limitations. It is the first study to date to investigate the quality and reliability of YouTube videos in a multitude of African languages widely spoken across the African continent. The sample size, a cumulative total of 562 videos, is larger than other similar studies.

The African continent is home to thousands of indigenous languages. Despite our attempt at selecting some of the widely spoken languages representatives of various regions in Africa, we could not accommodate several other languages with significant geographic and demographic representation across Africa. Qualitative studies with thematic analysis and behavioral studies are untapped territories that may provide a complimentary view with regards to YouTube’s role in health promotion, particularly, in Africa.

## 5. Conclusion

YouTube is an invaluable, easily accessible resource for information dissemination during health emergencies, as it has a broader and faster mainstream reach than on‐site community sensitization by appropriate health authorities. However, a concerning number of misleading videos abound on this platform, and consequently, before people access quality and reliable information during health emergencies, a vast majority of persons may be misinformed, compromising the adherence to proven public health measures. Fortunately, in our study, misleading videos accounted for a minor proportion. Although we showed some favorable levels of cumulative reliability in the YouTube videos assessed, we, however, found poor quality especially with regard to the information pertaining to screening, testing, and treatment. Academic medical centers, governments, and news agencies should improve the quality of their video content in these areas.

Various strategies need to be implemented to ensure a high caliber of medical information is available online to the public. Ultimately, this solution is the shared responsibility of the public, government, and YouTube itself. We implore all stakeholders to, therefore, join the global concerted efforts of health promotion and harnessing digital media, during this pandemic and beyond, in order to ultimately contain the spread of misinformation and achieve pandemic control [47].

## Disclosure

Earlier version of the manuscript was presented as a preprint in “COVID‐19 information on YouTube: analysis of quality and reliability of videos in eleven widely spoken languages across Africa” [46].

## Conflicts of Interest

The authors declare that they have no conflicts of interest.

## Authors’ Contributions

KN conceptualized the study, investigated the study, performed statistical analysis, wrote the first draft of the manuscript, and critically reviewed and edited the manuscript. KAP collected the data, wrote, reviewed, and edited the article. NU and AN assisted with methodology and reviewed and edited the article. OKW, OO, AO, and EW collected the data. EE, AH, SMG, ABM, NM and OX contributed equally towards data collection and review. All authors approved the final version of the manuscript.

## Supporting information


**Supplementary Materials** Supplementary Table 1: Set of criterion used for scoring within each MICI component. Supplementary Table 2: Modified DISCERN tool for evaluation of the reliability component.

## Data Availability

The dataset used to support the findings of this study can be obtained from the corresponding author upon reasonable request.
