# Supplementary material for: COVID‐19 Information on YouTube: Analysis of Quality and Reliability of Videos in Eleven Widely Spoken Languages across Africa
Source: Glob Health Epidemiol Genom. 2023 Jan 18;2023:1406035. doi: 10.1155/2023/1406035 (PMC9876664; doi:10.1155/2023/1406035)
Supplement: Supplementary file 1 — Supplementary Materials Supplementary Table 1: Set of criterion used for scoring within each MICI component. Supplementary Table 2: Modified DISCERN tool for evaluation of the reliability component. [file GHE3-2023-1406035-s001.docx]

**Supplementary Data**

| Prevalence | Each item is given 1 point if mentioned in the video. Maximum score of 5   1. Number of confirmed cases reported 2. Number of suspected cases reported 3. Number of deaths reported 4. Number of countries involved 5. Number/proportion of patients who are severely ill |
| --- | --- |
| Transmission | Each item is given 1 point if mentioned in the video. Maximum score of 5   1. Location of origin of virus 2. Zoonotic transmission (i.e. : contact with animals) 3. Human to human Transmission 4. Incubation period 5. Transmission route via droplets (include : precautionary measures of wearing mask , handwashing) |
| Signs and Symptoms | Each item is given 1 point if mentioned in the video. Maximum score of 5   1. Fever 2. Upper respiratory tract symptoms ( cough, sore throat, runny Nose) 3. Lower respiratory tract Symptoms (pneumonia)/ shortness of breath 4. Myalgia, arthralgia, lethargy 5. Diarrhea |
| Screening Testing | Each item is given 1 point if mentioned in the video. Maximum score of 5   1. Mentions there is a test available 2. Mentions the test uses respiratory secretion to test 3. Mentions that PCR can be used for identification 4. Shows how this test is done 5. Mentions criteria for testing/screening |
| Treatment / Outcome | Each item is given 1 point if mentioned in the video. Maximum score of 5   1. Mild symptoms can be self-resolving 2. Some patients becomes ill (mentions hospitalization, ICU admission ) 3. Can be dangerous, or lead to death 4. Treatment is supportive but HIV drugs are being used in some circumstances 5. Vaccination not currently available |

**Supplementary table 1: Set of criterion used for scoring within each MICI component^17^**

**Supplementary table 2 : Modified DISCERN tool for evaluation of reliability**^21^

| Item | Questions |
| --- | --- |
| 1 | Are the aims clear and achieved? |
| 2 | Are reliable sources of information used? (i.e. publication cited, the speaker is a specialist e.g. doctor, public health expert, infectious diseases expert, virologist, microbiologist) |
| 3 | Is the information presented balanced and unbiased? |
| 4 | Are additional sources of information listed for patient reference? |
| 5 | Are areas of uncertainty mentioned? |
